# Supplementary material for: Outcomes After VATS Single Versus Multiple Segmentectomy for cT1N0 Non-Small-Cell Lung Cancer
Source: Cancers (Basel). 2025 Nov 28;17(23):3814. doi: 10.3390/cancers17233814 (PMC12691515; doi:10.3390/cancers17233814)
Supplement: Supplementary file 1 [file cancers-17-03814-s001.zip › cancers-3977464-supplementary.pdf]

cancers

MDPI

Supplementary Materials: Outcomes After VATS Single Versus Multiple Segmentectomy for cT1N0 Non-Small-Cell Lung Cancer

Ye Tian, Edoardo Zanfrini, Etienne Abdelnour-Berchtold, Matthieu Zellweger, Jean Yannis Perentes, Thorsten Krueger and Michel Gonzalez

Table S1. Patients characteristics after PSM.

Variables	Total (*n* = 148)	SS (*n* = 74)	MS (*n* = 74)	*p*-Value
Age, Mean ± SD	67.2 ± 9.0	66.8 ± 9.6	67.6 ± 8.5	0.58
Gender, n (%)				0.869
Male	71 (48.0)	35 (47.3)	36 (48.6)	
Female	77 (52.0)	39 (52.7)	38 (51.4)	
Smoking, n (%)	127 (85.8)	63 (85.1)	64 (86.5)	0.814
BMI, Mean ± SD	25.9 ± 5.2	25.4 ± 5.3	26.4 ± 5.1	0.225
Previous Cancer, n (%)	68 (45.9)	30 (40.5)	38 (51.4)	0.187
CCI, Mean ± SD	5.2 ± 1.7	5.2 ± 1.6	5.3 ± 1.8	0.741
Comorbidity, n (%)	112 (75.7)	55 (74.3)	57 (77)	0.702
COPD, n (%)	62 (41.9)	31 (41.9)	31 (41.9)	1[a]
Hypertension, n (%)	82 (55.4)	40 (54.1)	42 (56.8)	0.741
Cardiovasular Disease, n (%)	42 (28.4)	22 (29.7)	20 (27)	0.715
Atrial Fibrillation, n (%)	16 (10.8)	7 (9.5)	9 (12.2)	0.597
Diabetes Mellitus, n (%)	29 (19.6)	13 (17.6)	16 (21.6)	0.534
Renal Insufficiency, n (%)	14 (9.5)	5 (6.8)	9 (12.2)	0.261
Median FEV1(%) (IQR)	84.0 (68.8, 100.0)	86.0 (67.2, 99.8)	84.0 (71.0, 100.8)	0.858
FEV1% Predicted, n (%)				0.615
FEV1% Predicted＜80%	59 (39.9)	31 (41.9)	28 (37.8)	
FEV1% Predicted≥80%	89 (60.1)	43 (58.1)	46 (62.2)	
Median DLCO (%) (IQR)	71.0 (59.0, 87.0)	71.5 (59.2, 84.0)	70.5 (57.8, 88.0)	0.854
DLCO% Predicted, n (%)				0.738
DLCO% Predicted＜80%	88 (59.5)	45 (60.8)	43 (58.1)	
DLCO% Predicted≥80%	60 (40.5)	29 (39.2)	31 (41.9)	
ASA, n (%)				1[b]
ASAI	1 (0.7)	0 (0)	1 (1.4)	
ASAII	67 (45.3)	34 (45.9)	33 (44.6)	
ASAIII	79 (53.4)	40 (54.1)	39 (52.7)	
ASAIV	1 (0.7)	0 (0)	1 (1.4)	

a:The p-value of 1.000 reflects perfect balance after PSM, rendering the hypothesis test non-significant. b:Fisher's exact test.

Table S2. Surgical characteristics and pathological results after PSM.

Variables	Total (*n* = 148)	SS (*n* = 74)	MS(*n* = 74)	*p*-Value
Lobe, n (%)				0.898
LLL	13 (8.8)	7 (9.5)	6 (8.1)	
LUL	63 (42.6)	31 (41.9)	32 (43.2)	
RLL	35 (23.6)	16 (21.6)	19 (25.7)	
RUL	37 (25.0)	20 (27)	17 (23)	
PETSUV, n (%)				0.319

SUVmax ＜ 2.5	64 (43.2)	29 (39.2)	35 (47.3)	
C.T_Ratio, n (%)				0.887
C/T Ratio ＜ 0.5	26 (17.6)	14 (18.9)	12 (16.2)	
0.5 ≤ C/T Ratio ＜ 1	46 (31.1)	22 (29.7)	24 (32.4)	
C/T Ratio = 1	76 (51.4)	38 (51.4)	38 (51.4)	
Tumor.Size.mm., Mean ± SD	15.4 ± 6.4	15.6 ± 6.3	15.2 ± 6.6	0.684
Margins..mm., Median (IQR)	12.0 (5.0, 20.0)	11.5 (6.0, 21.5)	12.0 (5.0, 15.8)	0.098
Ratio, Mean ± SD	1.1 ± 1.0	1.1 ± 1.0	1.1 ± 1.1	0.571
Tumor Location, n (%)				0.482
Central	48 (32.4)	22 (29.7)	26 (35.1)	
Peripheral	100 (67.6)	52 (70.3)	48 (64.9)	
Lymph Nodes Harvested, Mean ± SD	8.4 ± 5.0	8.3 ± 5.3	8.5 ± 4.7	0.793
Operative Time, Mean ± SD	118.3 ± 48.5	109.8 ± 40.8	126.9 ± 54.1	0.031
Conversion, n (%)	2 (1.4)	0 (0)	2 (2.7)	0.497[b]
Resection Status, n (%)				1[b]
R0	148 (100.0)	74 (100)	74 (100)	
Histology, n (%)				0.913
Adenocarcinoma	120 (81.1)	59 (79.7)	61 (82.4)	
Squamous cell carcinoma	17 (11.5)	9 (12.2)	8 (10.8)	
Others	11 (7.4)	6 (8.1)	5 (6.8)	
Pleural invasion, n (%)	15 (10.1)	12 (16.2)	3 (4.1)	0.014
T Stage, n (%)				0.539[b]
Tis	8 (5.4)	4 (5.4)	4 (5.4)	
T1a	35 (23.6)	16 (21.6)	19 (25.7)	
T1b	62 (41.9)	30 (40.5)	32 (43.2)	
T1c	26 (17.6)	12 (16.2)	14 (18.9)	
T2a	14 (9.5)	9 (12.2)	5 (6.8)	
T3	3 (2.0)	3 (4.1)	0 (0)	
N Stage, n (%)				0.866[b]
N0	138 (93.2)	70 (94.6)	68 (91.9)	
N1	3 (2.0)	1 (1.4)	2 (2.7)	
N2	6 (4.1)	3 (4.1)	3 (4.1)	
Nx	1 (0.7)	0 (0)	1 (1.4)	
Pathologic Stage, n (%)				0.945[b]
Stage0	8 (5.4)	4 (5.4)	4 (5.4)	
StageIA1	35 (23.6)	16 (21.6)	19 (25.7)	
StageIA2	58 (39.2)	28 (37.8)	30 (40.5)	
StageIA3	24 (16.2)	12 (16.2)	12 (16.2)	
StageIB	11 (7.4)	7 (9.5)	4 (5.4)	
StageIIB	6 (4.1)	4 (5.4)	2 (2.7)	
StageIIIA	6 (4.1)	3 (4.1)	3 (4.1)	
Adjuvant Chemotherapy, n (%)	22 (14.9)	15 (20.3)	7 (9.5)	0.065

b:Fisher's exact test; mm: millimeter.

Table S3. Post-operative outcomes after PSM.

Variables	Total (*n* = 148)	0 (*n* = 74)	1 (*n* = 74)	*p*-Value
Pneumonia, n (%)	12 (8.1)	3 (4.1)	9 (12.2)	0.071
Air.leak, n (%)	14 (9.5)	5 (6.8)	9 (12.2)	0.261
Empyema, n (%)				1[b]
Non	148 (100.0)	74 (100)	74 (100)	
Embolism, n (%)	1 (0.7)	0 (0)	1 (1.4)	1[b]
Atelectasis, n (%)	2 (1.4)	0 (0)	2 (2.7)	0.497[b]
Arrhythmia, n (%)	5 (3.4)	2 (2.7)	3 (4.1)	1[b]
Myocardial.infarction, n (%)				1[b]

Non	148 (100.0)	74 (100)	74 (100)	
Ileus, n (%)	2 (1.4)	1 (1.4)	1 (1.4)	1[b]
Colitis, n (%)	1 (0.7)	0 (0)	1 (1.4)	1[b]
Urosepsis, n (%)				1[b]
Non	148 (100.0)	74 (100)	74 (100)	
AKI, n (%)	6 (4.1)	1 (1.4)	5 (6.8)	0.209
TIA.Stroke, n (%)				1[b]
Non	148 (100.0)	74 (100)	74 (100)	
Reoperation, n (%)	4 (2.7)	1 (1.4)	3 (4.1)	0.62[b]
Median length of drainage days (IQR)	2.0 (1.0, 4.0)	1.0 (1.0, 3.0)	3.0 (1.2, 4.0)	0.005
Drain duration, n (%)				1[a]
＞5days	20 (13.5)	10 (13.5)	10 (13.5)	
Length of Stay Days, Median(IQR)	5.0 (4.0, 8.0)	4.0 (3.0, 6.0)	6.0 (5.0, 9.8)	<0.001
Recurrence, n (%)				0.79[b]
Local Recurrence	5 (3.4)	3 (4.1)	2 (2.7)	
Distant Recurrence	4 (2.7)	3 (4.1)	1 (1.4)	
Local combined Distant Recurrence	2 (1.4)	1 (1.4)	1 (1.4)	
Perioperative Mortality ≤30 days				1[b]
Non	148 (100.0)	74 (100)	74 (100)	

a:The p-value of 1.000 reflects perfect balance after PSM, rendering the hypothesis test non-significant. b:Fisher's exact test.

Table S4. Association between Surgical Strategy (SS vs. MS) and Survival Outcomes Before and After PSM.

Survival Outcome	Model	Group Comparison	Hazard Ratio (95% CI)	*P* -Value
Overall Survival (OS)	Unmatched Crude	MS vs. SS	0.56 (0.15–2.11)	0.393
	Multivariable Adjusted*	MS vs. SS	0.22 (0.05–1.07)	0.06
	Propensity Score Adjusted	MS vs. SS	0.22 (0.05–1.02)	0.053
	Propensity Score Matched	MS vs. SS	0.49 (0.09–2.74)	0.414
Disease-Free Survival (DFS)	Unmatched Crude	MS vs. SS	0.77 (0.26~2.27)	0.636
	Multivariable Adjusted*	MS vs. SS	0.42 (0.11~1.6)	0.204
	Propensity Score Adjusted	MS vs. SS	0.45 (0.12~1.62)	0.222
	Propensity Score Matched	MS vs. SS	0.74 (0.16~3.42)	0.7
Locoregional Recurrence-Free Survival (LRFS)	Unmatched Crude	MS vs. SS	1.27 (0.68~2.37)	0.453
	Multivariable Adjusted*	MS vs. SS	0.59 (0.26~1.35)	0.213
	Propensity Score Adjusted	MS vs. SS	0.65 (0.3~1.41)	0.276
	Propensity Score Matched	MS vs. SS	1.1 (0.47~2.6)	0.826

CI：Confidence Interval

Table S5. Covariates of PSM.

Item	Unmatched				Item	Matched			
n	SS	MS	SMD	SMD0.1	n	SS	MS	SMD	SMD0.1
	211	123		NA		74	74		NA
Age (mean (SD))	67.96 (9.35)	67.29 (9.30)	0.072	<0.1	Age (mean (SD))	66.82 (9.55)	67.65 (8.48)	0.091	<0.1
Smoking = 1 (%)	182 (86.3)	104 (84.6)	0.048	<0.1	Smoking = 1 (%)	63 (85.1)	64 (86.5)	0.039	<0.1

FEV1 (mean (SD))	85.36 (23.96)	81.43 (22.94)	0.168	>0.1	FEV1 (mean (SD))	83.14 (27.54)	84.05 (21.53)	0.037	<0.1
CCI (mean (SD))	5.30 (1.91)	5.33 (2.03)	0.011	<0.1	CCI (mean (SD))	5.19 (1.63)	5.28 (1.84)	0.054	<0.1
Comorbidity = 1 (%)	156 (73.9)	91 (74.0)	0.001	<0.1	Comorbidity (%)	55 (74.3)	57 (77.0)	0.063	<0.1
Tumor.Size.mm. (mean (SD))	14.33 (5.99)	15.46 (6.73)	0.176	>0.1	Tumor.Size.mm. (mean (SD))	15.62 (6.31)	15.19 (6.60)	0.067	<0.1
C.T_Ratio (%)			0.134	>0.1	C.T_Ratio (%)			0.081	<0.1
C/T Ratio < 0.5	45 (21.3)	20 (16.3)		NA	C/T Ratio < 0.5	14 (18.9)	12 (16.2)		NA
0.5 ≤ C/T Ratio < 1	61 (28.9)	36 (29.3)		NA	0.5 ≤ C/T Ratio < 1	22 (29.7)	24 (32.4)		NA
C/T Ratio = 1	105 (49.8)	67 (54.5)		NA	C/T Ratio = 1	38 (51.4)	38 (51.4)		NA
Loble (%)			1.245	>0.1	Loble (%)			0.127	>0.1
LLL	43 (20.4)	6 (4.9)		NA	LLL	7 (9.5)	6 (8.1)		NA
LUL	31 (14.7)	81 (65.9)		NA	LUL	31 (41.9)	32 (43.2)		NA
RLL	63 (29.9)	19 (15.4)		NA	RLL	16 (21.6)	19 (25.7)		NA
RUL	74 (35.1)	17 (13.8)		NA	RUL	20 (27.0)	17 (23.0)		NA
Tumor Location = 1 (%)	150 (71.1)	85 (69.1)	0.043	<0.1	Tumor Location = 1 (%)	52 (70.3)	48 (64.9)	0.116	>0.1

SMD: Standardized Mean Difference

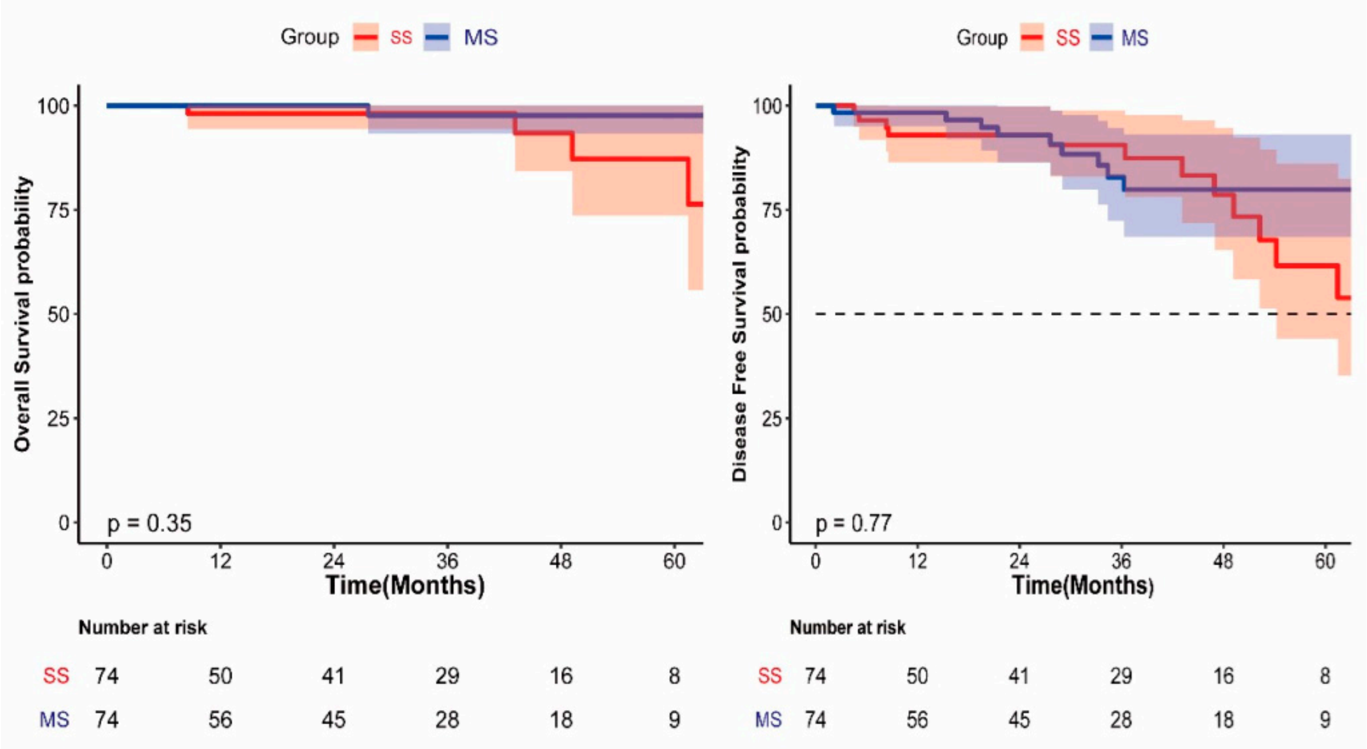

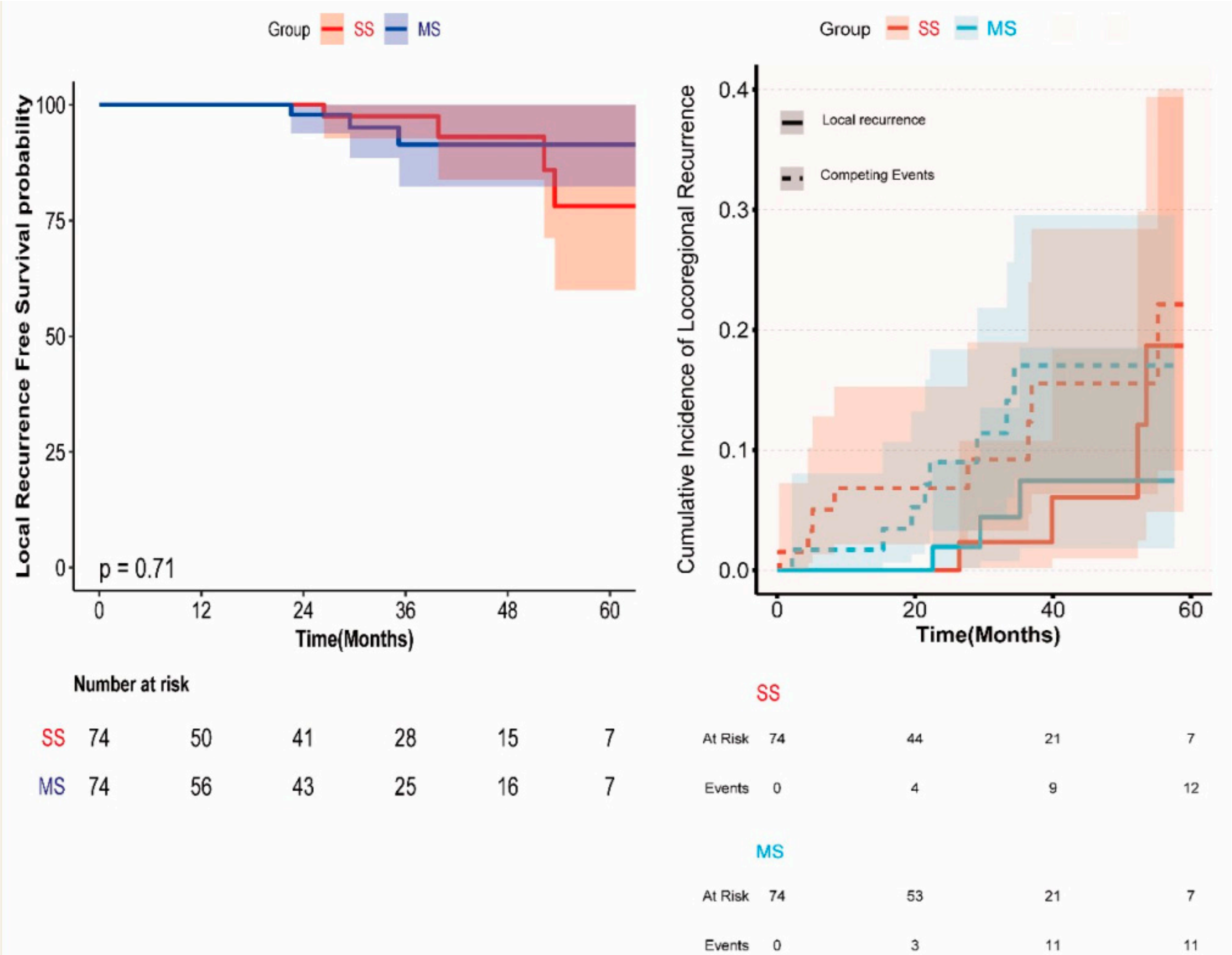

Figure S1. Propensity score-matched survival analysis comparing single segmentectomy (SS) and multiple segmentectomy (MS). Kaplan–Meier curves for OS, DFS, and LRFS, as well as the cumulative incidence of locoregional recurrence with competing risks, are shown for the matched cohort (74 SS vs. 74 MS).

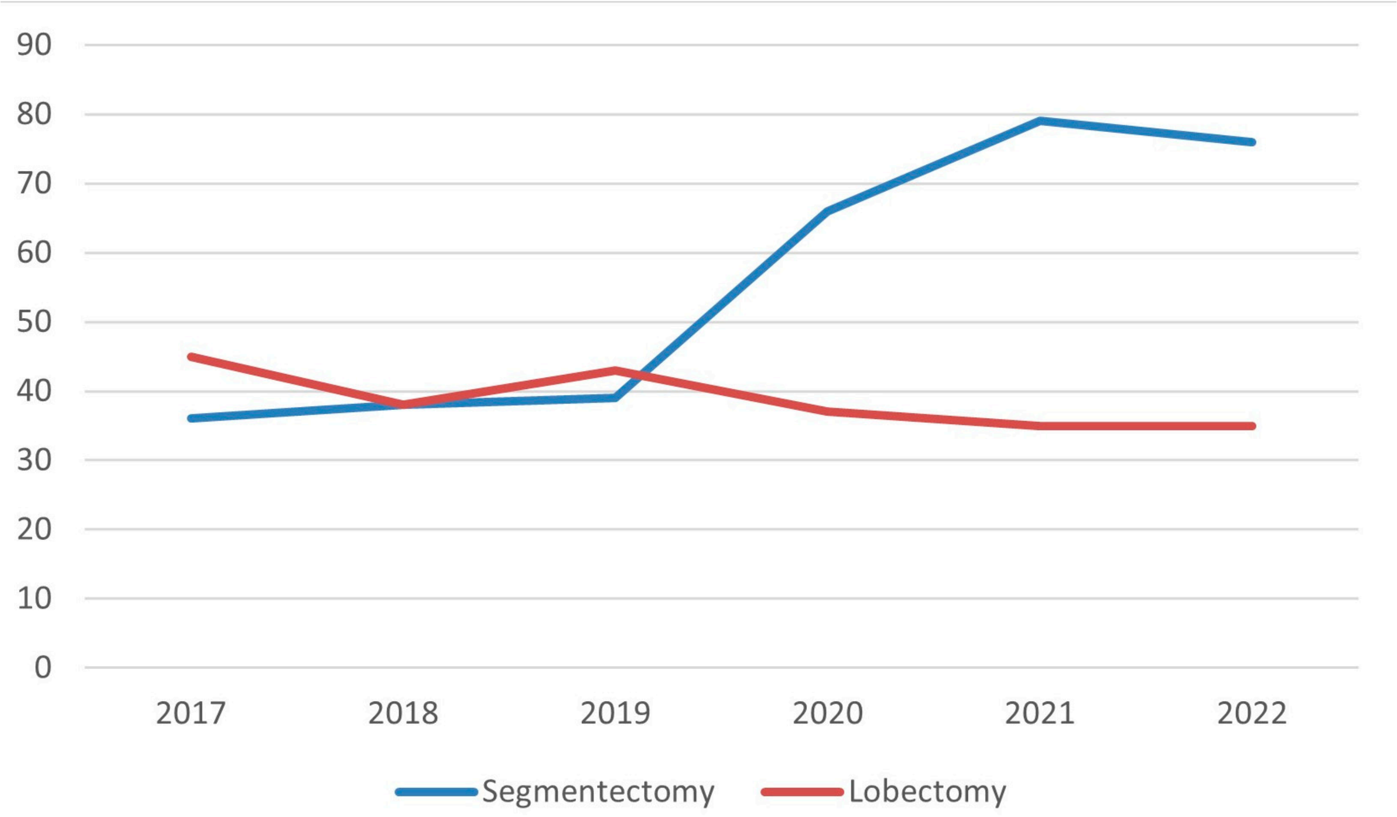

Figure S2. Annual number of segmentectomies and lobectomies performed at our institution from 2017 to 2022 for cT1N0 NSCLC.
